# Viral Pathogens in Oesophageal and Gastric Cancer

**DOI:** 10.3390/pathogens11040476

**Published:** 2022-04-15

**Authors:** Kishen Rajendra, Prateek Sharma

**Affiliations:** 1 School of Medicine, The International Medical University, Kuala Lumpur 57000, Malaysia; 2 Division of Gastroenterology and Hepatology, Veterans Affairs Medical Center, Kansas City, MO 64128, USA; psharma@kumc.edu; 3 School of Medicine, University of Kansas, Kansas City, MO 66160, USA

**Keywords:** viruses, oesophageal cancer, gastric cancer, human papillomavirus, Ebstein–Barr virus

## Abstract

Tumour virology was born with the discovery by Peyton Rous in 1911 of a filterable agent in chicken cellular extracts that caused neoplasia in healthy chickens. Universally, 20% of all human cancers have a viral aetiology. Viruses are involved at various stages of the carcinogenesis pathway, depending on the viral pathogen, and likely require co-factors. Multiple risk factors have been associated with oesophageal and gastric malignancy, including carcinogenic pathogens. These viruses and bacteria include human papillomavirus (HPV) [oesophageal cancer], Epstein–Barr virus (EBV) [proximal stomach cancer], and Helicobacter pylori (HP) [non-cardia stomach cancer]. Viruses such as EBV have been firmly established as causal for up to 10% of gastric cancers. HPV is associated with 13 to 35% of oesophageal adenocarcinoma but its role is unclear in oesophageal squamous cell carcinomas. The causal relationship between hepatitis B (HBV), cytomegalovirus (CMV), HPV, and John Cunningham (JCV) and gastric neoplasia remains indeterminate and warrants further study. The expression of viral antigens by human tumours offers preventive and therapeutic potential (including vaccination) and has already been harnessed with vaccines for HPV and HBV. Future goals include viral protein-based immunotherapy and monoclonal antibodies for the treatment of some of the subset of EBV and HPV-induced gastro-esophageal cancers.

## 1. Introduction

It is estimated that between 15% and 20% of all human cancers worldwide are caused by infectious agents [1,2]. Seven viruses [Epstein–Barr virus (EBV), hepatitis B virus, human papillomavirus (HPV), T-cell lymphotropic virus, hepatitis C virus, Kaposi’s sarcoma virus (KHSV)/human herpesvirus 8 (HHV-8), and Merkel cell polyomavirus] cause 12% of these cancers. Viruses are involved at various stages of the carcinogenesis pathway depending on the viral pathogen and likely require co-factors [e.g., smoking, contraceptives, nutrition, co-infection with herpesvirus and Chlamydia, human immunodeficiency virus (HIV) in cervical malignancy, alcohol, and aflatoxin in hepatocellular carcinoma] to trigger neoplasia [2,3,4]. This includes tumour initiation by integration of the viral DNA into the host genome causing upregulation of cellular oncogene expression, viral promotion of DNA damage, chromosomal instability, and dysregulation of cellular processes (proliferation, apoptosis, and replicative immortality) by viral proteins [2,5,6]. Some viruses e.g., HBV and HCV, cause hepatocellular carcinoma by indirect means i.e., chronic inflammation over decades supplemented by co-factors of aflatoxin and alcohol [7]. Another mechanism that is central to viral carcinogenesis is the interaction with the immune system with the consequent evolvement of immune evasion strategies. These include downregulation of the major histocompatibility complex (MHC), interfering with interferon action, molecular mimicry, and generation of escape mutants [2]. It is worth noting that many of the incriminated viruses in human cancers are ubiquitous in the general population, yet only a small minority develop a virally induced neoplasia.

Multiple risk factors have been associated with oesophageal and gastric malignancy, including carcinogenic pathogens. These viruses and bacteria include human papillomavirus (HPV) [oesophageal cancer], Epstein–Barr virus (EBV) [proximal stomach cancer], and Helicobacter pylori (HP) [non-cardia stomach cancer] [8,9,10]. The microbiome has also been incriminated in oesophageal and gastric diseases and will be discussed in another article in this Special Issue of Pathogens.

In this review, we discuss the published data linking viruses to oesophageal and gastric malignancy. We pay particular attention to the role of HPV and EBV in oesophageal and gastric cancers respectively. In addition, we discuss the role of lesser-known viruses e.g., John Cunningham Virus (JCV) and oesophageal and gastric tumours as well as the association between Hepatitis B and gastric neoplasia.

## 2. Oesophageal Cancer

Oesophageal cancer is the seventh most common cancer worldwide with a male preponderance (70%). There are an estimated 604,000 annual incident cases, and it is the sixth leading cause of cancer death with an estimated 544,000 deaths annually [11]. The two major histological subtypes are squamous cell carcinoma (88% cases) and adenocarcinoma (12% cases) [12]. Oesophageal squamous cell carcinoma (OSCC) constitutes the vast majority of oesophageal malignancies in the East. Oesophageal adenocarcinoma (OAC) predominates in the West and has been on an exponential trajectory upwards recently [13,14,15]. OSCC incidence varies significantly around the globe, and the highest incidence rates are in East Asia, Southern Africa, Eastern Africa, Northern Europe, and South-Central Asia [11].

## 3. OSCC

OSCC has a multifactorial aetiology depending on geographical location. In the West, excess alcohol and smoking is incriminated. Intriguingly, betel nut chewing in the Indian subcontinent and South-East Asia as well as drinking extremely hot tea in South America and Iran has been associated with OSCC. Other postulated risk factors include poverty, consuming pickled vegetables, and exposure to radiation [16,17,18]. In 1982, Syrjanen et al. first suggested an association between human papillomaviruses and oesophageal squamous cell carcinoma [19,20]. To this day, the association between HPV and OSCC remains contentious primarily due to the existence of positive and negative studies which shall be discussed in greater detail below.

## 4. HPV

HPV is a non-enveloped DNA virus that belongs to the papillomaviridae family and has more than 150 genotypes. Although it demonstrates tropism for squamous epithelium, low copy numbers of HPV DNA have been shown to be integrated in the glandular epithelium of cervical adenocarcinoma, OAC, and its precursor lesion, Barrett’s dysplasia (BD) [21,22,23]. They are classified into high-risk (e.g., HPV-16 and 18) and low-risk (HPV-6 and 11) based on their propensity to transform host cells and promote progression to cancer [24,25]. HPV carcinogenesis is best characterized in cervical squamous cancer whereby infection of the basal cell layer caused by micro-abrasions results in either a sub-clinical infection, or benign or malignant lesion [26]. Integration of HPV DNA into the host genome is thought to be a key step in carcinogenesis. Integration probably upregulates cellular oncogene expression (mainly E6 and E7) which may facilitate oncogenesis [4]. Integration of the viral genome disrupts the expression of the repressive E2 gene and thus facilitates the continued and abnormal expression of E6 and E7 oncoproteins [27]. E7 inhibits the retinoblastoma tumour suppressor protein (pRb) and causes proteosome-dependent degradation. E6 targets p53 degradation and upregulates the telomerase expression causing immortality of transformed cells [28]. HPV is now universally recognized as the causal agent in cervical cancer, oropharyngeal malignancy, and anal neoplasia [4,29,30].

## 5. HPV and OSCC

HPV-associated OSCC usually with genotypes 16 and 18 (0–78%) varies according to geographic location, study design, and detection methods employed [31,32]. Iran and Northern China with high overall incidence rates of OSCC report greater HPV tumour infection rates (32.8–63.6%) than countries with a lower incidence of OSCC, e.g., Europe and the USA with HPV tumour infection rates of 15.6% and 16.6%, respectively [33].

Another source for the anomaly in HPV prevalence rates in OSCC may relate to detection methods utilised to test for HPV. A systematic review conducted by Petrick et al. noted that HPV-OSCC prevalence varied depending on detection method; 7.6% for the Southern blot technique compared to 32.2% for HPV L1 serology. It should be noted that L1 is the major component of the HPV capsid and is indicative of cumulative exposure to HPV infection and does not necessarily denote an HPV-induced cancer. Nevertheless, the two most commonly used methods i.e., PCR and ISH, yielded similar HPV-OSCC rates [34]. Other systematic reviews and meta-analyses have resulted in contradictory data on this issue [35,36,37]. Type-specific primers result in higher HPV prevalence rates. These amplify short segments of DNA and are thus more sensitive than broad-spectrum primers that amplify longer fragments of DNA [36]. It has also been proposed that types of specimens used i.e., fresh-frozen versus formalin-fixed and other co-factors involved in oesophageal carcinogenesis have also been suggested as possible reasons for the discrepancies in HPV detection rates in OSCC [37].

Assessing transcriptional activity e.g., p16 or E6/E7 mRNA transcripts of HPV infection in OSCC, would be useful to determine if the virus has an aetiological role or otherwise in this malignancy. p16INK4A is widely considered a surrogate marker of hr-HPV infection in oropharyngeal cancers and cervical malignancy [38,39,40].

A systematic review revealed no significant correlation between HPV positive OSCC and p16 INK4A overexpression [41]. A Swedish study involving 204 patients with OSCC in whom 10% were HPV positive, found no difference in p16INK4A prevalence between HPV positive (24%) and negative tumours (16%) [42].

It is possible that the high p16 promoter methylation rate in OSCC may be responsible for the lack of p16 overexpression in HPV-positive cases [43].

High antibody titers to the HPV16 L1 (late capsid protein) have been associated with cancers of the cervix [44,45] and oropharynx [29,46].

Significant associations between OSCC and antibodies to E6 for HPV16 (OR = 1.89, 95% CI = 1.09–3.29, *p* = 0.023) and HPV6 (OR = 2.53, 95% CI = 1.51–4.25, *p* < 0.001) has been reported [47]. Nevertheless, a follow-up study by the same authors demonstrated no significant link between HPV and OSCC despite the serological associations [48].

The data on the prognostic role of HPV in OSCC are conflicting [31].

Studies have revealed either reduced overall survival (OS) in HPV-associated OSCC [49] or shown no survival differences between HPV-positive versus viral-negative oesophageal tumours [50,51,52]. On the contrary, Wang et al. reported that patients with HPV-DNA positive genotype 16 advanced OSCC, had a significantly better three-year survival than HPV-negative oesophageal malignancies (55% vs. 21%) as well as a superior response to chemoradiotherapy [53]. Another investigation revealed improved five-year OS and progression-free survival (PFS) in patients with HPV-positive OSCC as compared with HPV-negative osophageal cancer [54]. Similarly, Kumar and colleagues found that OSCC patients with p16-positive tumors subjected to neoadjuvant chemotherapy had better complete remission rates than the p16-negative group [55].

Overall, the role of HPV in OSCC is unclear. Well-designed, case-controlled studies using optimal viral detection methodology in appropriate tissue specimens whilst undertaking stringent steps to avoid contamination are required. Moreover, seeking viral transcriptional markers is essential in demonstrating a robust association (or otherwise) between HPV and OSCC.

## 6. OAC

OAC is one of the fastest growing and deadliest cancers in the Western World [13,56]. Traditionally, Barrett’s oesophagus (BO) has been considered the only visible precursor lesion for OAC. This ‘epidemic’ of OAC has occurred against a backdrop of progressive reduction in the risk estimate of malignancy associated with BO [57].

Risk factors for OAC include obesity, smoking, long-standing gastro-esophageal reflux disease (GORD), family history of GORD, BO, or OAC, older age, male sex, white race, persistent human papillomavirus infection, and possibly reduction in H. pylori infection rates [17,57,58]. Conversely, protective factors for OAC include H. pylori infection, use of non-steroidal anti-inflammatories, statins, and a diet high in fruit and vegetables [58].

## 7. HPV and OAC

**Figure 1:**
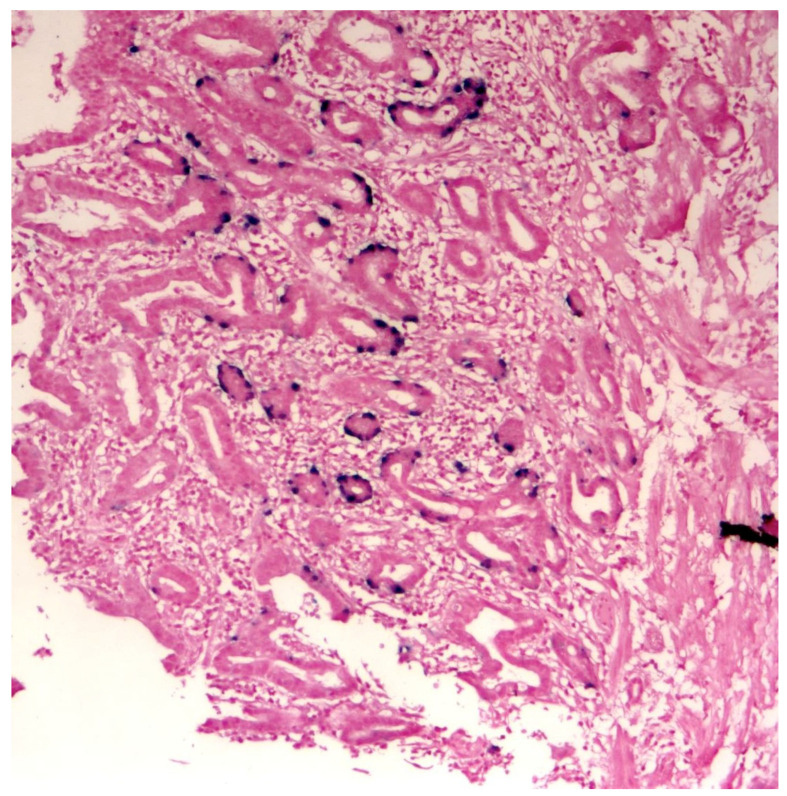
DNA in-situ hybridization demonstrating the presence of hr-HPV genome in oesophageal adenocarcinoma tissue. This clearly demonstrates HPV tropism for oesophageal glandular tissue. (Courtesy of Professor S. Rajendra.)

Systematic reviews have reported HPV prevalence rates of between 13% and 35% in patients with OAC [59,60]. The authors suggested that the lower prevalence rate may have been caused by small sample sizes and compromised detection methods [59]. Low HPV viral load further compounds the problem [23]. The discovery of a strong association of transcriptionally active high-risk human papillomavirus (hr-HPV) i.e., types 16 and 18 with a subset of Barrett’s dysplasia (BD) and OAC [55] may be relevant in explaining the significant rise of OAC since the 1970s. as has been the case with the epidemic of head and neck tumours, another viral associated cancer [61,62] (Figure 1).

Increasing hr-HPV viral load and integration status is associated with more severe disease in Barrett’s metaplasia–dysplasia–adenocarcinoma sequence [23]. Whole exome sequencing has revealed that HPV-positive OAC is biologically distinct to HPV-negative OAC suggesting a different mechanism of tumour formation [63]. From a molecular perspective, active human papillomavirus involvement in Barrett’s dysplasia and oesophageal adenocarcinoma is characterized by wild-type p53, upregulation of p16INK4A, and downregulation of pRb [64].

Rajendra et al. have reported that HPV-positive Barrett’s high-grade dysplasia and OAC have an improved survival compared with viral-negative osophageal tumours [65]. They demonstrated superior disease-free survival (DFS) for HPV and biomarkers for transcriptionally active viruses i.e., E6, E7 mRNA, and high p16 expression but not p53. The authors postulated that the survival benefit was derived from a three-fold reduction in distant metastasis and possibly better loco-regional control in HPV+ as compared to HPV- patients. This translated to a 2.7-times lower mortality from OAC in the viral positive group. The mean duration of overall survival was again significantly improved in the HPV + group as compared with the HPV- category [65]. This is analogous to HPV-induced head and neck squamous cell carcinomas (HNSCC) being a distinct subset with a more favourable prognosis compared to viral negative oropharyngeal malignancies [66,67]. In a follow-up study, the Sydney group demonstrated that HPV status conferred a significant effect modification in association with pRb, cyclin D_1_ MCM, and Ki-67 in regard to survival, disease relapse/progression, metastatic spread, and disease-specific death [68]. Patients with osophageal tumors, which were HPV+/pRB_low,_ had a significantly improved DFS compared with HPV-/pRB_high_ on univariable analysis (HR, 0.29; 95% CI, 0.10–0.82, *p* = 0.02) and also after adjusting for age, gender, BMI, ever smoked, excess alcohol, and resection margin (HR, 0.33; 95% CI, 0.12–0.93, adjusted *p* = 0.04). Similarly, HPV+/CD1_low_ conferred a significantly favorable DFS (HR, 0.26; 95% CI, 0.09–0.76, adjusted *p* = 0.01) as did HPV+/MCM _high_ (HR, 0.27; 95% CI, 0.09–0.78, adjusted *p* = 0.02) [68]. In regard to OS, HPV had a significant interaction only with CD1 _low_, conferring a significantly improved survival (HR, 0.38; 95% CI, 0.15–0.94, adjusted *p* = 0.04) [13].

Negative association studies on HPV and BD/OAC have been reported, and the results may have been adversely affected by poor tissue classification, suboptimal testing methods, small sample sizes, racial and geographical variations, and the use of metaplastic tissue, which is not associated with the virus [69,70,71,72]. Utilisation of formalin-fixed tissue specimens greater than 10 years old with a consequent risk of DNA/RNA degradation may also explain the discrepancy [73]. Overall though, it is now clearly recognized that a subset of BD and OAC (approximately 25%) is associated with hr-HPV genotypes 16 and 18.

## 8. EBV

EBV was the first human tumour virus identified in cells cultured from Burkitt’s lymphoma [74]. It is a DNA herpesvirus (HHV-4) with a 168 to 184 kbp genome and infects more than 90% of the world’s population causing mostly asymptomatic infections [75,76]. It is a lymphotropic and epitheliotropic infection and consists of two subtypes, EBV-1 and EBV-2 (based on sequence differences in the Epstein–Barr nuclear antigen) [77]. Type 1 infections are the predominant strain worldwide (Asia, Europe, and the Americas), and type 2 strains are found mainly in Africa and New Guinea [78]. It is responsible for 1.5% of all human cancers worldwide, mainly lymphomas and nasopharyngeal cancers but also other non-lymphoid malignancies, including leiomyosarcomas and gastric malignancy [7]. Therefore EBV has been classified as a Class I carcinogen by the International Agency for Research on Cancer [79].

EBV primarily infects the epithelium of the oropharynx, then replicates and spreads to B cells establishing a latent infection that is responsible for many human malignancies [80]. Depending on the viral gene expression pattern, the infection can be classified into three latency types (types I, II, and III) and will be discussed in greater detail below under the subheading of EBV and gastric cancer. The latent genomes express six EBV-encoded nuclear antigens and three latent membrane proteins (LMPs) i.e., EBC-encoded small RNA (EBER) and non-transcribed BART (BamHI-A region rightward transcript) [81]. The persistence of the viral genome in malignant cells and the expression of limited latent genes as well as co-factors (e.g., co-infections and comorbidities) are important elements in carcinogenesis.

## 9. EBV and OSCC

The first report of EBV DNA detection in OSCC was published in 1996 by Jenkins et al. [82]. Using microdissected tumour samples they found 5/60 oesophageal tumour samples to be positive as well as 1/16 OSCC cell lines. Mizobuchi et al. assessed 41 surgical specimens as well as 12 cell lines of OSCC for EBV EBNA-1 gene via PCR and found none [83]. Another Japanese study by Yanai et al. detected no EBER (EBV encoded RNA) -1-positive cell using ISH in 36 surgically resected OSCC [84]. Likewise, a study from Thailand investigated 104 surgically resected OSCC and found no EBER-positive cancer cells by ISH [85]. Wang et al. analysed 51 paraffin-embedded OSCC samples (9 well differentiated, 31 moderately differentiated, and 11 poorly differentiated tumours) from a high-risk area in Northern China for EBER via ISH and PCR amplification for EBV *Bam*HI W fragment and returned a total negative result [86]. Conversely, in a study from Taiwan, EBV DNA was detected by PCR in 11/31 (35.5%) of patients with OSCC [87]. These results were confirmed by EBER detection by ISH. Awerkiew et al. investigated the presence of EBV DNA (PCR) and EBER transcripts (ISH) in 72 OSCC and 40 OAC from Germany as well as 43 OSCC from Russia. They found that 34% of OSCC and 26% of OAC contained EBV DNA but no EBER transcripts in tumour nuclei. Nevertheless, in the 24 EBV DNA-positive cases, EBER transcripts were detected in the nuclei of tumour infiltrating lymphocytes in 7 OSCC and 1 OAC. Given there was no persistence of EBV in tumour cells, the authors rightly concluded a negative association [88]. Hong et al. reported another negative study whereby no EBV DNA was identified in 30 OSCC and 2 OAC cell lines [89]. The most compelling positive report was by Wu et al. whereby they analysed 164 oesophageal cancers (151 OSCC and 13 undifferentiated cancers) for EBV. EBV EBER and LMP-1 proteins were identified in 10 (6.1%) tumour specimens by both ISH and IHC and were confined to poorly differentiated squamous cell carcinomas or undifferentiated carcinomas with intense lymphoid infiltration [90].

The contradictory data (analogous to that noted in HPV and OSCC) is due to a combination of geographical, racial, and detection technique differences. Furthermore, it is unclear if strict measures were undertaken to prevent contamination. The use of old formalin-fixed tissue specimens can result in RNA degradation and hence a higher false-negative outcome with the use of ISH. Nevertheless, it seems a small minority of OSCC is associated with EBV.

## 10. EBV and OAC

No association of EBV with OAC has been reported. In a study of 162 OAC, 92 cardia adenocarcinoma and 89 gastric adenocarcinoma, EBER transcripts were found in 0 (0%) of OAC, 3 of cardia adenocarcinoma (3.3%), and 8 of the gastric adenocarcinoma (8.9%) samples [91]. In another study of 465 oesophageal and gastric adenocarcinoma resected specimens (118 OAC, 73 GOJ adenocarcinomas, and 274 gastric adenocarcinomas). EBER detection via ISH was 0 in OAC, 2 in GOJ (2.7%), and 12 in gastric cancer (4.4%) [92].

## 11. Other Viruses and Oesophageal Cancer

John Cunningham virus (JC) is a small DNA virus that causes progressive multifocal leukoencephalopathy; it is a rare, often fatal disease. Only one notable study exists in relation to the detection of JCV DNA in both oesophageal cancer tissue (5/5, 100%) and normal oesophageal epithelium (11/13, 85%). The detection of viral proteins i.e., JCV T antigen and agnoprotein, which signifies a productive infection was only found in carcinomatous tissue (53% and 42%, respectively) and not benign oesophageal specimens (0%) [93]. The authors conclude that the results suggest a potential role of JCV in the development of upper gastrointestinal tract malignancies. Moreover, The International Agency for Cancer Research Monograph Working Group considers JCV as possibly carcinogenic to humans (belonging to group 2B), on the premise that there is sufficient evidence in experimental animals [94].

Wu et al. assessed 164 OSCC surgical specimens from Shantou, China and found HSV DNA and protein of HSV I and II in 31.7% of moderately differentiated samples [90]. Another study from the Shantou found a prevalence rate of HSV (I) of 30%, which was significantly higher than normal oesophageal mucosa [95].

Conversely, Chang et al. reported a negative association of HSV and OSCC using IHC [96].

## 12. Gastric Cancer

Gastric cancer ranks as the fifth-highest malignancy by incidence in the world with just over a million new cases in 2020. It is also the fourth-deadliest tumour with 769,000 deaths worldwide [11]. The highest incidence is in South-Central Asia (Iran, Afghanistan, Turkmenistan, and Kyrgyzstan); East Asia (Mongolia and Japan); and Eastern Europe. Gastric cancer is generally classified according to anatomical sub-sites i.e., cardia and non-cardia malignancies with differing risk factors [11]. Chronic helicobacter pylori infection is the major cause of gastric malignancies (75%), predominantly in the distal stomach [97]. Other risk factors include smoking, excess alcohol intake, and preservatives (e.g., nitrates and nitrites in processed meats can be converted into nitrosoamines in the stomach, which can be carcinogenic). Low fruit consumption and high intake of processed meats are possibly associated with an increased risk of gastric cancer [98].

Cardia cancers are generally associated with obesity and GORD [99,100,101,102]. H. Pylori is not considered a risk factor for this type of cancer. There may even be an inverse relationship of H. pylori infection and cardia tumours [103,104]. It is postulated that the bacteria-induced corpus atrophy reduces gastric acid secretion and thus decreased GORD.

Another classification of gastric cancers is based on the Cancer Genome Atlas Consortium (TCGA) molecular subtypes of (1) Epstein–Barr virus-positive tumours, (2) microsatellite unstable tumours, (3) genomically stable tumours, and (4) tumours with chromosomal instability [105].

## 13. EBV and Gastric Cancer

Epstein–Barr virus is associated with 8.4% of gastric cancers (predominantly adenocarcinomas). Proximal cancers i.e., cardia and corpus, were much more likely to be EBV positive (13.6% and 13.1%, respectively) as compared with antral tumours (5.2%) [106]. The TCGA genomic analysis revealed approximately 9% of gastric cancers are EBV positive [107]. Burke et al. documented the first EBV-positive gastric neoplasia a lymphoepithelial carcinoma in 1990 using PCR [107]. Nevertheless, lymphoepithelial-like (LEL) tumours only account for 1% to 4% of all gastric cancers but 90% are EBV-positive [106]. EBV-positive gastric tumours as compared with virus-negative lesions tend to be a younger cohort, have a greater male preponderance, have a stronger association with smoking, be more likely of distal location, have reduced lymph node metastases, and have a more favourable prognosis [108,109,110,111]. From a molecular perspective, EBV-positive gastric cancers have fewer chromosomal aberrations and somatic mutations, extensive DNA hypermethylation, increased mutations of PIK3CA, ARID1A and BCOR, and amplifications/high expression of JAK-2, PD-L1, and PD-L2 [105,112,113,114].

**Figure 2:**
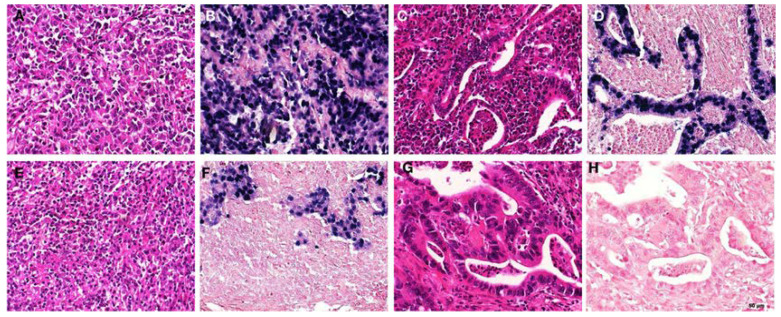
EBER staining patterns and morphology in gastric carcinomas. (**A**,**B**) “Lymphoepithelioma-like” morphology; EBER positive [(**A**) HE; (**B**) EBER-ISH], (**C**,**D**) “intestinal type” morphology, EBER positive [(**C**) HE; (**D**) EBER-ISH]; (**E**,**F**) “lymphoepithelioma-like” morphology; EBER negative in the tumour, but positive in accompanying lymphocytic infiltrate [(**E**) HE; (**F**) EBER-ISH]; (**G**,**H**) “intestinal-type” morphology, EBER negative [(**G**) HE; (**H**) EBER-ISH] (EBER, EBV-encoded small RNAs; ISH, in situ hybridization). https://www.researchgate.net/figure/Examples-of-EBER-staining-patterns-and-morphology-in-gastric-carcinomas-A-B_fig1_274722785 (accessed on 6 March 2022) [92].

EBER-1 is the most used method to detect EBV infections in tissue specimens [9]. It is present in abundance in latent virus-infected cells. Normally, EBER-1 is detected in almost all the nuclei of the virus-positive tumour (Figure 2). It is now accepted that EBV-associated gastric carcinoma is a result of the monoclonal proliferation of the virus-infected cell [115].

EBV entry into epithelial cells (facilitated by possibly local inflammation and/or pre-malignant changes of the gastric epithelium) involves multiple interactions between viral and host proteins, which is poorly understood [116]. It is postulated that virus acquisition is via lytic reactivation of a B cell reservoir [9,111]. The viral gene expression pattern at presentation is either latency type I (EBNA1, EBER1, EBER2, BamHI-A) or, in up to 50% of cases, latency I/II with the addition of LMP2A [117]. Expression of both lytic genes (that promote active replication of virus with the release of new progeny viral particles upon lysis of host cell) and latent genes (responsible for the quiescent, non-replicating phase) is essential in gastric carcinogenesis [118].

Methylation of both viral and host DNA is important in the development and progression of EBV-induced gastric malignancy. Methylation of viral DNA controls the expression of both lytic and latent genes whereas methylation of host cell DNA inactivates the tumour suppressor genes [119]. LMP2A is responsible for DNA methyltransferase activation via phosphorylation of STA3 [120]. Another key driver of cancer is the EBV-induced multiple epigenetic abnormalities in host cells [121]. Tet Methylcytosine Dioxygenase 2 (TET2) downregulation is necessary for DNA methylation in EBV-induced gastric cancer [122]. As such, several tumour suppressor genes e.g., p16, p14, APC, and TP73, become methylated [123,124,125]. EBNA1 and EBNA3C also inhibit the p53 tumour suppressor gene transcription and facilitate its degradation [126]. This may account for the scarcity of mutations in the TP53 gene [116].

## 14. HPV and Gastric Cancer

There have been contradictory studies on the role of HPV in GC. Both positive and negative association publications are abound [127,128,129,130]. Nevertheless, a recent meta-analysis of fourteen studies investigating the prevalence of HPV in 901 gastric cancer patients and 1205 controls revealed a pooled prevalence rate of 23.6% in the former. There was a significant association between HPV infection and the risk of gastric malignancy (OR = 1.53, 95% CI 1.00–2.33, *p* = 0.002) [131].

## 15. Other Viruses and Gastric Cancer

A systemic review/meta-analysis of nine studies that assessed 692 gastric cancer versus 664 controls found a pooled JCV prevalence of 35.6%. There was a significant correlation between this virus and gastric malignancy (OR = 2.28, 95% CI 1.14–4.56) [131]. The presence of JCV transforming antigen was detected in all included studies as it is important in carcinogenesis. Moreover, JCV infection has been correlated with activation of B-catenin, wild-type p53, allelic losses, and aberrant methylation of multiple genes [116,132,133,134,135,136,137].

The same meta-analysis investigated the role of HBV (hepatitis B virus) and 53, 396 stomach cancer patients as compared to 325, 458 controls. This revealed a pooled prevalence rate of 7.6% in gastric neoplasia. A significant association was detected between HBV and the risk of gastric malignancy (OR = 1.56, 95% CI 1.18–2.07) [131]. Likewise, the CMV detection rate was analysed in the same systematic review involving 265 gastric malignancy and 417 non-cancer controls, and significantly the pooled OR was 2.25, 95% CI 1.14–4.43 [131].

## 16. Conclusions

The role of viruses in carcinogenesis has been heavily debated for many years.

The mere detection of viral DNA, RNA, or proteins is inadequate in demonstrating causality. Nevertheless, viruses such as EBV have been firmly established as causal for up to 10% of gastric cancers. The role of HPV in a significant minority of OAC is gaining momentum and acceptance given the weight of positive association studies [58,138,139], but more contentious for OSCC. The causal relationship between HBV, CMV, HPV, JCV, and gastric neoplasia remains indeterminate and warrants further investigation. The expression of viral antigens by human tumours presents preventive and therapeutic potential and has already been harnessed with vaccines for HPV and HBV. Future goals include viral protein-based immunotherapy, monoclonal antibodies, and small molecule inhibitors [2].

## References

[1] De Martel C., Georges D., Bray F., Ferlay J., Clifford G.M. (2020). Global burden of cancer attributable to infections in 2018: A worldwide incidence analysis. Lancet Glob. Health.

[2] White M.K., Pagano J.S., Khalili K. (2014). Viruses and human cancers: A long road of discovery of molecular paradigms. Clin. Microbiol. Rev..

[3] Maxwell J.H., Khan S., Ferris R.L., Fakhry C., D’Souza G. (2015). The molecular biology of HPV-related head and neck cancer. HPV and Head and Neck Cancers.

[4] Samoff E., Koumans E.H., Markowitz L.E., Sawyer M.K., Swan D., Papp J.R., Black C.M., Unger E.R. (2005). Association of chlamydia trachomatis with persistence of high-risk types of human papillomavirus in a cohort of female adolescents. Am. J. Epidemiol..

[5] Dürst M., Croce C.M., Gissmann L., Schwarz E., Huebner K. (1987). Papillomavirus sequences integrate near cellular oncogenes in some cervical carcinomas. Proc. Natl. Acad. Sci. USA.

[6] Moore P.S., Chang Y. (2010). Why do viruses cause cancer? Highlights of the first century of human tumour virology. Nat. Rev. Cancer.

[7] Yeh F.S., MoYen R.C. (1985). Risk factors for hepatocellular carcinoma in Guanxi, People’s Republic of China. Natl. Cancer Inst. Monogr..

[8] Al-Haddad S., El-Zimaity H., Hafezi-Bakhtiari S., Rajendra S., Streutker C.J., Vajpeyi R., Wang B. (2014). Infection and esophageal cancer. Ann. N. Y. Acad. Sci..

[9] Nishikawa J., Iizasa H., Yoshiyama H., Shimokuri K., Kobayashi Y., Sasaki S., Nakamura M., Yanai H., Sakai K., Suehiro Y. (2018). Clinical importance of Epstein-Barr virus-associated gastric cancer. Cancers.

[10] Parsonnet J., Friedman G.D., Vandersteen D.P., Chang Y., Vogelman J.H., Orentreich N., Sibley R.K. (1991). Helicobacter pylori infection and the risk of gastric carcinoma. N. Engl. J. Med..

[11] Sung H., Ferlay J., Siegel R.L., Laversanne M., Soerjomataram I., Jemal A., Bray F. (2021). Global cancer statistics 2020: Globocan estimates of incidence and mortality worldwide for 36 cancers in 185 countries. CA A Cancer J. Clin..

[12] Arnold M., Soerjomataram I., Ferlay J., Forman D. (2015). Global incidence of oesophageal cancer by histological subtype in 2012. Gut.

[13] Pohl H., Welch H.G. (2005). The role of overdiagnosis and reclassification in the marked increase of esophageal adenocarcinoma incidence. J. Natl. Cancer Inst..

[14] Pohl H., Sirovich B., Welch H.G. (2010). Esophageal adenocarcinoma incidence: Are we reaching the peak?. Cancer Epidemiol. Prev. Biomarkers Prev..

[15] Lagergren J., Mattsson F. (2011). No further increase in the incidence of esophageal adenocarcinoma in Sweden. Int. J. Cancer.

[16] Bray F., Ferlay J., Soerjomataram I., Siegel R.L., Torre L.A., Jemal A. (2018). Global cancer statistics 2018: Globocan estimates of incidence and mortality worldwide for 36 cancers in 185 countries. CA A Cancer J. Clin..

[17] Thun M.J., Linet M.S., Cerhan J.R., Haiman C., Schottenfeld D. (2018). Schottenfeld and Fraumeni Cancer Epidemiology and Prevention.

[18] Abnet C.C., Arnold M., Wei W.Q. (2018). Epidemiology of esophageal squamous cell carcinoma. Gastroenterology.

[19] Syrjanen K., Pyrhonen S., Aukee S., Koskela E. (1982). Squamous cell papilloma of the esophagus: A tumour probably caused by human papilloma virus (HPV). Diagn. Histopathol..

[20] Syrjanen K.J. (1982). Histological changes identical to those of condylomatous lesions found in esophageal squamous cell carcinomas. Arch. Geschwulstforsch..

[21] Pirog E.C., Kleter B., Olgac S., Bobkiewicz P., Lindeman J., Quint W.G., Richart R.M., Isacson C. (2000). Prevalence of human papillomavirus DNA in different histological subtypes of cervical adenocarcinoma. Am. J. Pathol..

[22] Cooper K., Herrington C.S., Lo E.S., Evans M.F., McGee J.O. (1992). Integration of human papillomavirus types 16 and 18 in cervical adenocarcinoma. J. Clin. Pathol..

[23] Wang B., Rajendra S., Pavey D., Sharma P., Merrett N., Wu X., Snow E.T., Kumbhari V., Ball M.J., Robertson I.K. (2013). Viral load and integration status of high-risk human papillomaviruses in the Barrett’s metaplasia-dysplasia-adenocarcinoma sequence. Am. J. Gastroenterol..

[24] De Villiers E.M., Fauquet C., Broker T.R., Bernard H.U., Zur Hausen H. (2004). Classification of papillomaviruses. Virology.

[25] Van Doorslaer K., Tan Q., Xirasagar S., Bandaru S., Gopalan V., Mohamoud Y., Huyen Y., McBride A.A. (2013). The papillomavirus episteme: A central resource for papillomavirus sequence data and analysis. Nucleic Acids Res..

[26] Schiffman M., Castle P.E., Jeronimo J., Rodriguez A.C., Wacholder S. (2007). Human papillomavirus and cervical cancer. Lancet.

[27] Pett M.R., Alazawi W.O., Roberts I., Dowen S., Smith D.I., Stanley M.A., Coleman N. (2004). Acquisition of high-level chromosomal instability is associated with integration of human papillomavirus type 16 in cervical keratinocytes. Cancer Res..

[28] Moody C.A., Laimins L.A. (2010). Human papillomavirus oncoproteins: Pathways to transformation. Nat. Rev. Cancer.

[29] Herrero R., Castellsague X., Pawlita M., Lissowska J., Kee F., Balaram P., Rajkumar T., Sridhar H., Rose B., Pintos J. (2003). Human papillomavirus and oral cancer: The International Agency for Research on Cancer multicenter study. J. Natl. Cancer Inst..

[30] Carter J.J., Madeleine M.M., Shera K., Schwartz S.M., Cushing-Haugen K.L., Wipf G.C., Porter P., Daling J.R., McDougall J.K., Galloway D.A. (2001). Human papillomavirus 16 and 18 L1 serology compared across anogenital cancer sites. Cancer Res..

[31] Ludmir E.B., Stephens S.J., Palta M., Willett C.G., Czito B.G. (2015). Human papillomavirus tumor infection in esophageal squamous cell carcinoma. J. Gastrointest. Oncol..

[32] Syrjänen K. (2013). Geographic origin is a significant determinant of human papillomavirus prevalence in oesophageal squamous cell carcinoma: Systematic review and meta-analysis. Scand. J. Infect. Dis..

[33] Hardefeldt H.A., Cox M.R., Eslick G.D. (2014). Association between human papillomavirus (HPV) and oesophageal squamous cell carcinoma: A meta-analysis. Epidemiol. Infect..

[34] Petrick J., Wyss A., Butler A., Cummings C., Sun X., Poole C., Smith J., Olshan A.F. (2014). Prevalence of human papillomavirus among oesophageal squamous cell carcinoma cases: Systematic review and meta-analysis. Br. J. Cancer.

[35] Syrjanen K.J. (2002). HPV infections and oesophageal cancer. J. Clin. Pathol..

[36] Yong F., Xudong N., Lijie T. (2013). Human papillomavirus types 16 and 18 in esophagus squamous cell carcinoma: A meta-analysis. Ann. Epidemiol..

[37] Liyanage S.S., Rahman B., Ridda I., Newall A.T., Tabrizi S.N., Garland S.M., Segelov E., Seale H., Crowe P.J., Moa A. (2013). The aetiological role of human papillomavirus in oesophageal squamous cell carcinoma: A meta-analysis. PLoS ONE.

[38] Kumar B., Cordell K.G., Lee J.S., Worden F.P., Prince M.E., Tran H.H., Wolf G.T., Urba S.G., Chepeha D.B., Teknos T.N. (2008). EGFR, p16, HPV Titer, Bcl-xL and p53, sex, and smoking as indicators of response to therapy and survival in oropharyngeal cancer. J. Clin. Oncol..

[39] Ishikawa M., Fujii T., Saito M., Nindl I., Ono A., Kubushiro K., Tsukazaki K., Mukai M., Nozawa S. (2006). Overexpression of p16 INK4a as an indicator for human papillomavirus oncogenic activity in cervical squamous neoplasia. Int. J. Gynecol. Cancer.

[40] Lakshmi S., Rema P., Somanathan T. (2009). P16ink4a is a surrogate marker for high-risk and malignant cervical lesions in the presence of human papillomavirus. Pathobiology.

[41] Michaelsen S.H., Larsen C.G., Von Buchwald C. (2014). Human papillomavirus shows highly variable prevalence in esophageal squamous cell carcinoma and no significant correlation to p16INK4a overexpression: A systematic review. J. Thorac. Oncol..

[42] Löfdahl H.E., Du J., Näsman A., Andersson E., Rubio C.A., Lu Y., Ramqvist T., Dalianis T., Lagergren J., Dahlstrand H. (2012). Prevalence of human papillomavirus (HPV) in oesophageal squamous cell carcinoma in relation to anatomical site of the tumour. PLoS ONE.

[43] Zhou C., Li J., Li Q. (2017). CDKN2A methylation in esophageal cancer: A meta-analysis. Oncotarget.

[44] Naucler P., Chen H.C., Persson K., You S.L., Hsieh C.Y., Sun C.A., Dillner J., Chen C.J. (2007). Seroprevalence of human papillomaviruses and Chlamydia trachomatis and cervical cancer risk: Nested case-control study. J. Gen. Virol..

[45] Sitas F., Urban M., Stein L., Beral V., Ruff P., Hale M., Patel M., O’Connell D., Qin Yu X., Verzijden A. (2007). The relationship between anti-HPV-16 IgG seropositivity and cancer of the cervix, anogenital organs, oral cavity and pharynx, oesophagus and prostate in a black South African population. Infect. Agents Cancer.

[46] D’Souza G., Kreimer A.R., Viscidi R., Pawlita M., Fakhry C., Koch W.M., Westra W.H., Gillison M.L. (2007). Case-control study of human papillomavirus and oropharyngeal cancer. N. Engl. J. Med..

[47] Sitas F., Egger S., Urban M.I., Taylor P.R., Abnet C.C., Boffetta P., O’Connell D.L., Whiteman D.C., Brennan P., Malekzadeh R. (2012). InterSCOPE study: Associations between esophageal squamous cell carcinoma and human papillomavirus serological markers. J. Natl. Cancer Inst..

[48] Halec G., Schmitt M., Egger S., Abnet C., Babb De Villiers C., Dawsey S., Flechtenmacher C., Gheit T., Hale M., Holzinger D. (2016). Mucosal alpha-papillomaviruses are not associated with esophageal squamous cell carcinomas: Lack of mechanistic evidence from South Africa, China and Iran and from a world-wide meta-analysis. Int. J. Cancer.

[49] Furihata M., Ohtsuki Y., Ogoshi S., Takahashi A., Tamiya T., Ogata T. (1993). Prognostic significance of human papillomavirus genomes (type-16,-18) and aberrant expression of p53 protein in human esophageal cancer. Int. J. Cancer.

[50] Dreilich M., Bergqvist M., Moberg M., Brattström D., Gustavsson I., Bergström S., Wanders A., Hesselius P., Wagenius G., Gyllensten U. (2006). High-risk human papilloma virus (HPV) and survival in patients with esophageal carcinoma: A pilot study. BMC Cancer.

[51] Antonsson A., Green A.C., Mallitt K.A., O’Rourke P.K., Pandeya N., Pawlita M., Waterboer T., Neale R.E. (2010). Prevalence and stability of antibodies to 37 human papillomavirus types--a population-based longitudinal study. Virology.

[52] Hippeläinen M., Eskelinen M., Lipponen P.K., Chang F., Syrjänen K. (1993). Mitotic activity index, volume corrected mitotic index and human papilloma-virus suggestive morphology are not prognostic factors in carcinoma of the oesophagus. Anticancer Res..

[53] Wang W.L., Wang Y.C., Lee C.T., Chang C.Y., Lo J.L., Kuo Y.H., Hsu Y.C., Mo L.R. (2015). The impact of human papillomavirus infection on the survival and treatment response of patients with esophageal cancers. J. Dig. Dis..

[54] Cao F., Zhang W., Zhang F., Han H., Xu J., Cheng Y. (2014). Prognostic significance of high-risk human papillomavirus and p16(INK4A) in patients with esophageal squamous cell carcinoma. Int. J. Clin. Exp. Med..

[55] Kumar R., Ghosh S.K., Verma A.K., Talukdar A., Deka M.K., Wagh M., Bahar H.M., Tapkire R., Chakraborty K.P., Kannan R.R. (2015). P16 expression as a surrogate marker for HPV infection in esophageal squamous cell carcinoma can predict response to neo-adjuvant chemotherapy. Asian Pac. J. Cancer Prev..

[56] Eloubeidi M.A., Mason A.C., Desmond R.A., El-Serag H.B. (2003). Temporal trends (1973–1997) in survival of patients with esophageal adenocarcinoma in the United States: A glimmer of hope?. Am. J. Gastroenterol..

[57] Rajendra S., Wang B., Snow E.T., Sharma P., Pavey D., Merrett N., Ball M.J., Brain T., Fernando R., Robertson I.K. (2013). Transcriptionally active human papillomavirus is strongly associated with Barrett’s dysplasia and esophageal adenocarcinoma. Am. J. Gastroenterol..

[58] Spechler S.J., Souza R.F. (2014). Barrett’s esophagus. N. Engl. J. Med..

[59] Kunzmann A.T., Graham S., McShane C.M., Doyle J., Tommasino M., Johnston B., Jamison J., James J.A., McManus D., Anderson L.A. (2017). The prevalence of viral agents in esophageal adenocarcinoma and Barrett’s esophagus: A systematic review. Eur. J. Gastroenterol. Hepatol..

[60] Li X., Gao C., Yang Y., Zhou F., Li M., Jin Q., Gao L. (2014). Systematic review with meta-analysis: The association between human papillomavirus infection and oesophageal cancer. Aliment. Pharmacol. Ther..

[61] Nasman A., Attner P., Hammarstedt L., Du J., Eriksson M., Giraud G., Ahrlund-Richter S., Marklund L., Romanitan M., Lindquist D. (2009). Incidence of human papillomavirus (HPV) positive tonsillar carcinoma in Stockholm, Sweden: An epidemic of viral-induced carcinoma?. Int. J. Cancer.

[62] Chaturvedi A.K., Engels E.A., Pfeiffer R.M., Hernandez B.Y., Xiao W., Kim E., Jiang B., Goodman M.T., Sibug-Saber M., Cozen W. (2011). Human papillomavirus and rising oropharyngeal cancer incidence in the United States. J. Clin. Oncol..

[63] Rajendra S., Wang B., Merrett N., Sharma P., Humphris J., Lee H.C., Wu J. (2016). Genomic analysis of HPV-positive versus HPV-negative oesophageal adenocarcinoma identifies a differential mutational landscape. J. Med. Genet..

[64] Rajendra S., Yang T., Xuan W., Sharma P., Pavey D., Lee C.S., Le S., Collins J., Wang B. (2017). Active human papillomavirus involvement in Barrett’s dysplasia and oesophageal adenocarcinoma is characterized by wild-type p53 and aberrations of the retinoblastoma protein pathway. Int. J. Cancer.

[65] Rajendra S., Xuan W., Merrett N., Sharma P., Sharma P., Pavey D., Yang T., Santos L.D., Sharaiha O., Pande G. (2018). Survival rates for patients with Barrett high-grade dysplasia and esophageal adenocarcinoma with or without human papillomavirus infection. JAMA Netw. Open.

[66] Ragin C.C., Taioli E. (2007). Survival of squamous cell carcinoma of the head and neck in relation to human papillomavirus infection: Review and meta-analysis. Int. J. Cancer.

[67] O’Rorke M.A., Ellison M.V., Murray L.J., Moran M., James J., Anderson L.A. (2012). Human papillomavirus related head and neck cancer survival: A systematic review and meta-analysis. Oral Oncol..

[68] Rajendra S., Sharma P., Gautam S.D., Saxena M., Kapur A., Sharma P., Merrett N., Yang T., Santos L.D., Pavey D. (2020). Association of biomarkers for human papillomavirus with survival among adults with Barrett high-grade dysplasia and esophageal adenocarcinoma. JAMA Netw. Open.

[69] El-Serag H.B., Hollier J.M., Gravitt P., Alsarraj A., Younes M. (2013). Human papillomavirus and the risk of Barrett’s esophagus. Dis. Esophagus.

[70] Rai N., Jenkins G.J., McAdam E., Hibbitts S.J., Fiander A.N., Powell N.G. (2008). Human papillomavirus infection in Barrett’s oesophagus in the UK: An infrequent event. J. Clin. Virol..

[71] Iyer A., Rajendran V., Adamson C.S., Peng Z., Cooper K., Evans M.F. (2011). Human papillomavirus is detectable in Barrett’s esophagus and esophageal carcinoma but is unlikely to be of any etiologic significance. J. Clin. Virol..

[72] Rajendra S., Wang B. (2016). Human papillomavirus not detected in esophageal adenocarcinoma tumor specimens-Letter. Cancer Epidemiol..

[73] Antonsson A., Knight L., Whiteman D.C. (2016). Human papillomavirus not detected in esophageal adenocarcinoma tumor specimens. Cancer Epidemiol..

[74] Epstein M.A., Achong B.G., Barr Y.M. (1964). Virus particles in cultured lymphoblasts from Burkitt’s lymphoma. Lancet.

[75] Baer R., Bankier A.T., Biggin M.D., Deininger P.L., Farrell P.J., Gibson T.J., Hatfull G., Hudson G.S., Satchwell S.C., Séguin C. (1984). DNA sequence and expression of the B95-8 Epstein-Barr virus genome. Nature.

[76] Henle G., Henle W., Clifford P., Diehl V., Kafuko G.W., Kirya B.G., Klein G., Morrow R.H., Munube G.M., Pike P. (1969). Antibodies to Epstein-Barr virus in Burkitt’s lymphoma and control groups. J. Natl. Cancer Inst..

[77] Sample J., Young L., Martin B., Chatman T., Kieff E., Rickinson A., Kieff E. (1990). Epstein-Barr virus types 1 and 2 differ in their EBNA-3A, EBNA-3B, and EBNA-3C genes. J. Virol..

[78] Palser A.L., Grayson N.E., White R.E., Corton C., Correia S., Ba Abdullah M.M., Watson S.J., Cotten M., Arrand J.R., Murray P.G. (2015). Genome diversity of Epstein-Barr virus from multiple tumor types and normal infection. J. Virol..

[79] International Agency for Research on Cancer (1997). IARC monographs on the evaluation of carcinogenic risks to humans. Epstein-Barr Virus and Kaposi’s Sarcoma Herpesvirus/Human Herpesvirus 8.

[80] Sixbey J.W., Nedrud J.G., Raab-Traub N., Hanes R.A., Pagano J.S. (1984). Epstein-Barr virus replication in oropharyngeal epithelial cells. N. Engl. J. Med..

[81] Young L.S., Rickinson A.B. (2004). Epstein-Barr virus: 40 years on. Nat. Rev. Cancer.

[82] Jenkins T.D., Nakagawa H., Rustgi A.K. (1996). The association of Epstein-Barr virus DNA with esophageal squamous cell carcinoma. Oncogene.

[83] Mizobuchi S., Sakamoto H., Tachimori Y., Kato H., Watanabe H., Terada M. (1997). Absence of human papillomavirus-16 and -18 DNA and Epstein-Barr virus DNA in esophageal squamous cell carcinoma. Jpn. J. Clin. Oncol..

[84] Yanai H., Hirano A., Matsusaki K., Kawano T., Miura O., Yoshida T., Okita K., Shimizu N. (2003). Epstein-Barr virus association is rare in esophageal squamous cell carcinoma. Int. J. Gastrointest. Cancer.

[85] Sunpaweravong S., Mitarnun W., Puttawibul P. (2005). Absence of Epstein-Barr virus in esophageal squamous cell carcinoma. Dis. Esophagus.

[86] Wang J., Noffsinger A., Stemmermann G., Fenoglio-Preiser C. (1999). Esophageal squamous cell carcinomas arising in patients from a high-risk area of North China lack an association with Epstein-Barr virus. Cancer Epidemiol. Prev. Biomark..

[87] Wang L.S., Chow K.C., Wu Y.C., Li W.Y., Huang M.H. (1999). Detection of Epstein-Barr virus in esophageal squamous cell carcinoma in Taiwan. Am. J. Gastroenterol..

[88] Awerkiew S., Zur Hausen A., Baldus S.E., Hölscher A.H., Sidorenko S.I., Kutsev S.I., Pfister H.J. (2005). Presence of Epstein-Barr virus in esophageal cancer is restricted to tumor infiltrating lymphocytes. Med. Microbiol. Immunol..

[89] Hong T., Shimada Y., Kano M., Kaganoi J., Uchida S., Komoto I., Yamabe H., Imamura M. (2000). The Epstein-Barr virus is rarely associated with esophageal cancer. Int. J. Mol. Med..

[90] Wu M.Y., Wu X.Y., Zhuang C.X. (2005). Detection of HSV and EBV in esophageal carcinomas from a high-incidence area in Shantou China. Dis. Esophagus.

[91] Sarbia M., Zur Hausen A., Feith M., Geddert H., Von Rahden B.H.A., Langer R., Von Weyhern C., Siewert J.R., Höfler H., Stein H.J. (2005). Esophageal (Barrett’s) adenocarcinoma is not associated with Epstein-Barr virus infection: An analysis of 162 cases. Int. J. Cancer.

[92] Genitsch V., Novotny A., Seiler C.A., Kröll D., Walch A., Langer R. (2015). Epstein–Barr virus in gastro-esophageal adenocarcinomas–Single center experiences in the context of current literature. Front. Oncol..

[93] Del Valle L., White M.K., Enam S., Piña Oviedo S., Bromer M.Q., Thomas R.M., Parkman H.P., Khalili K. (2005). Detection of JC virus DNA sequences and expression of viral T antigen and agnoprotein in esophageal carcinoma. Cancer.

[94] Bouvard V., Baan R.A., Grosse Y., Lauby-Secretan B., El Ghissassi F., Benbrahim-Tallaa L., Guha N., Straif K. (2012). Carcinogenicity of malaria and of some polyomaviruses. Lancet Oncol..

[95] Zhang D.H., Zhang Q.Y., Hong C.Q., Chen J.Y., Shen Z.Y., Zhu Y. (2011). Prevalence and association of human papillomavirus 16, Epstein-Barr virus, herpes simplex virus-1 and cytomegalovirus infection with human esophageal carcinoma: A case-control study. Oncol. Rep..

[96] Chang F., Syrjänen S., Shen Q., Cintorino M., Santopietro R., Tosi P., Syrjänen K. (2000). Evaluation of HPV, CMV, HSV and EBV in esophageal squamous cell carcinomas from a high-incidence area of China. Anticancer. Res..

[97] De Martel C., Forman D., Plummer M. (2013). Gastric cancer: Epidemiology and risk factors. Gastroenterol. Clin. N. Am..

[98] World Cancer Research Fund/American Institute for Cancer Research The Continuous Update Project Expert Report 2018. Diet, Nutrition, Physical Activity and Cancer: Colorectal Cancer. http://dietandcancerreport.org.

[99] Corley D.A., Kubo A., Zhao W. (2008). Abdominal obesity and the risk of esophageal and gastric cardia carcinomas. Cancer Epidemiol. Prev. Biomark..

[100] Merry A.H., Schouten L.J., Goldbohm R.A., Van Den Brandt P.A. (2007). Body mass index, height and risk of adenocarcinoma of the oesophagus and gastric cardia: A prospective cohort study. Gut.

[101] Derakhshan M.H., Malekzadeh R., Watabe H., Yazdanbod A., Fyfe V., Kazemi A., Rakhshani N., Didevar R., Sotoudeh M., Zolfeghari A.A. (2008). Combination of gastric atrophy, reflux symptoms and histological subtype indicates two distinct aetiologies of gastric cardia cancer. Gut.

[102] Wu A.H., Tseng C.C., Bernstein L. (2003). Hiatal hernia, reflux symptoms, body size, and risk of esophageal and gastric adenocarcinoma. Cancer.

[103] Kamangar F., Dawsey S.M., Blaser M.J., Perez-Perez G.I., Pietinen P., Newschaffer C.J., Abnet C.C., Albanes D., Virtamo J., Taylor P.R. (2006). Opposing risks of gastric cardia and noncardia gastric adenocarcinomas associated with Helicobacter pylori seropositivity. J. Natl. Cancer Inst..

[104] Chow W.H., Blaser M.J., Blot W.J., Gammon M.D., Vaughan T.L., Risch H.A., Perez-Perez G.I., Schoenberg J.B., Stanford J.L., Rotterdam H. (1998). An inverse relation between cagA+ strains of Helicobacter pylori infection and risk of esophageal and gastric cardia adenocarcinoma. Cancer Res..

[105] Cancer Genome Atlas Research Network (2014). Comprehensive molecular characterization of gastric adenocarcinoma. Nature.

[106] Murphy G., Pfeiffer R., Camargo M.C., Rabkin C.S. (2009). Meta-analysis shows that prevalence of Epstein-Barr virus-positive gastric cancer differs based on sex and anatomic location. Gastroenterology.

[107] Burke A.P., Yen T.S., Shekitka K.M., Sobin L.H. (1990). Lymphoepithelial carcinoma of the stomach with Epstein-Barr virus demonstrated by polymerase chain reaction. Mod. Pathol..

[108] Lee J.-H., Kim S.-H., Han S.-H., An J.-S., Lee E.-S., Kim Y.-S. (2009). Clinicopathological and molecular characteristics of Epstein–Barr virus-associated gastric carcinoma: A meta-analysis. J. Gastroenterol. Hepatol..

[109] Camargo M.C., Koriyama C., Matsuo K., Kim W.H., Herrera-Goepfert R., Liao L.M., Yu J., Carrasquilla G., Sung J.J., Alvarado-Cabrero I. (2014). Case-case comparison of smoking and alcohol risk associations with Epstein-Barr virus-positive gastric cancer. Int. J. Cancer.

[110] Camargo M.C., Kim W.H., Chiaravalli A.M., Kim K.M., Corvalan A.H., Matsuo K., Yu J., Sung J.J., Herrera-Goepfert R., Meneses-Gonzalez F. (2014). Improved survival of gastric cancer with tumour Epstein-Barr virus positivity: An international pooled analysis. Gut.

[111] Shannon-Lowe C., Rickinson A. (2019). The global landscape of EBV-associated tumors. Front. Oncol..

[112] Cristescu R., Lee J., Nebozhyn M., Kim K.M., Ting J.C., Wong S.S., Liu J., Yue Y.G., Wang J., Yu K. (2015). Molecular analysis of gastric cancer identifies subtypes associated with distinct clinical outcomes. Nat. Med..

[113] Dong M., Wang H.Y., Zhao X.X., Chen J.N., Zhang Y.W., Huang Y., Xue L., Li H.G., Du H., Wu X.Y. (2016). Expression and prognostic roles of PIK3CA, JAK2, PD-L1, and PD-L2 in Epstein-Barr virus-associated gastric carcinoma. Hum. Pathol..

[114] Wang K., Kan J., Yuen S.T., Shi S.T., Chu K.M., Law S., Chan T.L., Kan Z., Chan A.S., Tsui W.Y. (2011). Exome sequencing identifies frequent mutation of ARID1A in molecular subtypes of gastric cancer. Nat. Genet..

[115] Imai S., Koizumi S., Sugiura M., Tokunaga M., Uemura Y., Yamamoto N., Tanaka S., Sato E., Osato T. (1994). Gastric carcinoma: Monoclonal epithelial malignant cells expressing Epstein-Barr virus latent infection protein. Proc. Natl. Acad. Sci. USA.

[116] Palrasu M., Zaika E., El-Rifai W., Que J., Zaika A.I. (2021). Role of bacterial and viral pathogens in gastric carcinogenesis. Cancers.

[117] Ribeiro J., Oliveira C., Malta M., Sousa H. (2017). Epstein-Barr virus gene expression and latency pattern in gastric carcinomas: A systematic review. Future Oncol..

[118] Borozan I., Zapatka M., Frappier L., Ferretti V. (2018). Analysis of Epstein-Barr virus genomes and expression profiles in gastric adenocarcinoma. J. Virol..

[119] Kang G.H., Lee S., Kim W.H., Lee H.W., Kim J.C., Rhyu M.G., Ro J.Y. (2002). Epstein-barr virus-positive gastric carcinoma demonstrates frequent aberrant methylation of multiple genes and constitutes CpG island methylator phenotype-positive gastric carcinoma. Am. J. Pathol..

[120] Hino R., Uozaki H., Murakami N., Ushiku T., Shinozaki A., Ishikawa S., Morikawa T., Nakaya T., Sakatani T., Takada K. (2009). Activation of DNA methyltransferase 1 by EBV latent membrane protein 2A leads to promoter hypermethylation of PTEN gene in gastric carcinoma. Cancer Res..

[121] Kaneda A., Matsusaka K., Aburatani H., Fukayama M. (2012). Epstein-Barr virus infection as an epigenetic driver of tumorigenesis. Cancer Res..

[122] Namba-Fukuyo H., Funata S., Matsusaka K., Fukuyo M., Rahmutulla B., Mano Y., Fukayama M., Aburatani H., Kaneda A. (2016). TET2 functions as a resistance factor against DNA methylation acquisition during Epstein-Barr virus infection. Oncotarget.

[123] Geddert H., Zur Hausen A., Gabbert H.E., Sarbia M. (2011). EBV-infection in cardiac and non-cardiac gastric adenocarcinomas is associated with promoter methylation of p16, p14 and APC, but not hMLH1. Cell. Oncol..

[124] He D., Zhang Y.W., Zhang N.N., Zhou L., Chen J.N., Jiang Y., Shao C.K. (2015). Aberrant gene promoter methylation of p16, FHIT, CRBP1, WWOX, and DLC-1 in Epstein-Barr virus-associated gastric carcinomas. Med. Oncol..

[125] Ushiku T., Chong J.M., Uozaki H., Hino R., Chang M.S., Sudo M., Rani B.R., Sakuma K., Nagai H., Fukayama M. (2007). P73 gene promoter methylation in Epstein-Barr virus-associated gastric carcinoma. Int. J. Cancer.

[126] Yi F., Saha A., Murakami M., Kumar P., Knight J.S., Cai Q., Choudhuri T., Robertson E.S. (2009). Epstein-Barr virus nuclear antigen 3C targets p53 and modulates its transcriptional and apoptotic activities. Virology.

[127] Bozdayi G., Dinc B., Avcikucuk H., Turhan N., Altay-Kocak A., Ozkan S., Ozin Y., Bostanci B. (2019). Is human papillomavirus and helicobacter pylori related in gastric lesions?. Clin. Lab..

[128] Zeng Z.-M., Luo F.-F., Zou L.-X., He R.-Q., Pan D.-H., Chen X., Xie T.-T., Li Y.-Q., Peng Z.-G., Chen G. (2016). Human papillomavirus as a potential risk factor for gastric cancer: A meta-analysis of 1917 cases. OncoTargets Ther..

[129] De Souza C.R.T., Almeida M.C.A., Khayat A.S., Da Silva E.L., Soares P.C., Chaves L.C., Burbano R.M.R. (2018). Association between Helicobacter pylori, Epstein-Barr virus, human papillomavirus and gastric adenocarcinomas. World J. Gastroenterol..

[130] Snietura M., Waniczek D., Piglowski W., Kopec A., Nowakowska-Zajdel E., Lorenc Z., Muc-Wierzgon M. (2014). Potential role of human papilloma virus in the pathogenesis of gastric cancer. World J. Gastroenterol..

[131] Wang H., Chen X.-L., Liu K., Bai D., Zhang W.-H., Chen X.-Z., Hu J.-K. (2020). Associations between gastric cancer risk and virus infection other than Epstein-Barr virus: A systematic review and meta-analysis based on epidemiological studies. Clin. Transl. Gastroenterol..

[132] Kutsuna T., Zheng H., Abdel-Aziz H.O., Murai Y., Tsuneyama K., Furuta I., Takano Y. (2008). High JC virus load in tongue carcinomas may be a risk factor for tongue tumorigenesis. Virchows Arch..

[133] Izi S., Youssefi M., Rahmani F., Roshan N.M., Yari A., Avval F.Z. (2018). Detection of JC Polyomavirus tumor antigen in gastric carcinoma: A report from Iran. Iran. J. Microbiol..

[134] Ksiaa F., Ziadi S., Mokni M., Korbi S., Trimeche M. (2010). The presence of JC virus in gastric carcinomas correlates with patient’s age, intestinal histological type and aberrant methylation of tumor suppressor genes. Mod. Pathol..

[135] Yamaoka S., Yamamoto H., Nosho K., Taniguchi H., Adachi Y., Sasaki S., Arimura Y., Imai K., Shinomura Y. (2009). Genetic and epigenetic characteristics of gastric cancers with JC virus T-antigen. World J. Gastroenterol..

[136] Murai Y., Zheng H.C., Abdel Aziz H.O., Mei H., Kutsuna T., Nakanishi Y., Tsuneyama K., Takano Y. (2007). High JC virus load in gastric cancer and adjacent non-cancerous mucosa. Cancer Sci..

[137] Shin S.K., Li M.S., Fuerst F., Hotchkiss E., Meyer R., Kim I.T., Goel A., Boland C.R. (2006). Oncogenic T-antigen of JC virus is present frequently in human gastric cancers. Cancer.

[138] Gibson M.K., Tanabe K.K., Goldberg R.M., Savarese D.M.F. (2022). Epidemiology and Pathobiology of Esophageal Cancer.

[139] Li S., Luk H.Y., Xia C., Chen Z., Chan P.K.S., Boon S.S. (2021). Oesophageal carcinoma: The prevalence of DNA tumour viruses and therapy. Tumour Virus Res..

